# Risk Factors for Obstetric Fistula in Western Uganda: A Case Control Study

**DOI:** 10.1371/journal.pone.0112299

**Published:** 2014-11-17

**Authors:** Justus Kafunjo Barageine, Nazarius Mbona Tumwesigye, Josaphat K. Byamugisha, Lars Almroth, Elisabeth Faxelid

**Affiliations:** 1 Department of Obstetrics and Gynecology, School of medicine, Makerere University College of Health Sciences/Mulago National Referral Hospital, Kampala, Uganda; 2 Department of Public Health Sciences, Global Health (IHCAR), Karolinska Institutet, Stockholm, Sweden; 3 Department of epidemiology and biostatistics, School of Public Health, Makerere University College of Health Sciences, Kampala, Uganda; University of Aberdeen, United Kingdom

## Abstract

**Introduction:**

Two million women worldwide are living with genital fistula with an annual incidence of 50,000–100,000 women. Risk factors for obstetric fistula are context bound. Studies from other countries show variation in the risk factors for obstetric fistula. This study was conducted to identify risk factors for obstetric fistula in western Ugandan context.

**Methods:**

A case control study comparing background factors of women with obstetric fistula (cases) and women without fistula (controls) was conducted in western Uganda. Data was collected using face-to-face interviews. Univariate, bivariate and multivariate analysis was conducted using Stata 12.

**Results:**

Altogether, 420 respondents (140 cases and 280 controls) participated in the study. Duration of labour was used to form the product terms when assessing for interaction and confounding since it was one the most significant factors at bivariate level with a narrow confidence interval and was hence considered the main predictor. After adjusting for interaction and confounding, significant risk factors associated with development of obstetric fistula in western Uganda were: Caesarean section (adjusted odds ratio [AOR]  = 13.30, 95% CI  = 6.74–26.39), respondent height of 150 cm or less (AOR  = 2.63, 95% CI  = 1.35–5.26), baby weight of 3.5 kg or more (AOR  = 1.52, 95% CI  = 1.15–1.99), prolonged labour (AOR  = 1.06, 95% CI  = 1.04–1.08. A quarter of the fistulas had resulted from iatrogenic complication during caesarean section. Compared to no education, post primary level of education was protective against obstetric fistula (AOR  = 0.31, 95% CI  = 0.13–0.72) and there was no difference between respondents without education and those with primary level education.

**Conclusions:**

Surgeons contribute to a big proportion (25%) of fistula cases hence caesarean section being a risk factor in this region. Other risk factors include; prolonged labour, weight of the baby of 3.5 kg or more, respondent height of 150 cm or less (short stature), and low or no education are risk factors for obstetric fistula in western Ugandan.

## Introduction

Globally, 99 per cent of the 289,000 maternal deaths are in low-income countries [1], [2]. For each maternal death, 15 to 30 other women are left with serious morbidities including genital fistula, a preventable condition [3]. A genital fistula is an abnormal hole between the urinary tract or rectum and the genital tract, through which urine and/or faeces continually leak. A genital fistula is referred to as obstetric if it results from the process of labour or its management [4], [5]. When a fistula is from the urinary system it is referred to as Urogenital fistula [4]. About two million women worldwide have genital fistula with an annual incidence of 50,000 to 100,000 cases [6], [7]. In sub-Saharan Africa, 33,000 new fistula cases occur each year and this high incidence may be attributed to obstetric care services being unavailable, inaccessible, underutilized or of low quality [8], [9]. In Uganda, the national prevalence of fistula is two per cent among women aged 15–49 years and the prevalence is highest in the western region where one in 25 women has ever been affected [10], [11].

Most of the genital fistulas in low resource countries follow prolonged and neglected obstructed labour [6], [12], [13]. Other obstetric causes include destructive deliveries, caesarean section with or without hysterectomy, and symphysiotomy [6], [14]–[16]. Social, cultural and health system factors contribute to the prevalence of obstetric fistula in low-income countries [17], [18]. Fistula surgeons have observed that these factors are context bound and include: lack of emergency obstetric care, child marriage associated with early pregnancy, severe forms of female genital cutting, gender discrimination, poverty, malnutrition, and poor health services [19]–[21]. Maternal age less than 20 years has been cited to increase the risk of development of fistula but this seems to vary from country to country with a mean age of 22 years in Ethiopia and 28 in Nigeria [16],[22],[23]. Other risk factors that have been cited include prime parity, prolonged labour, stillbirth delivery, and poor socio-economic status [15], [16], [24], [25]. In Ethiopia, more than 60 per cent of the women with obstetric fistula are primiparas, with average labour duration of 3.9 days and 92 per cent are illiterate [16], [25]. Similar trends of illiteracy have been observed in Nigeria [26] and in East Africa [27].

Lack of access to appropriate emergency obstetric care has been highlighted as one of the main risk factor for obstetric fistula [21], [22]. Access to appropriate obstetric care is compounded by poverty and the dynamics in the health care system including the cost of a caesarean section and availability of service providers [28]. Other risk factors such as young mothers, first pregnancy and cultural practices have not been found to cause fistula in high-income countries where emergency obstetric care is available [4], [28]. These factors might, however, be important in low-income countries where some women delay in seeking care and thus don't obtain timely emergency obstetric care [4], [28]. The reasons for not seeking skilled care vary according to context such as educational level, socio-economic status, culture as well as accessibility to functioning health care facilities [4], [26], [29].

Currently, no epidemiological study has documented risk factors for obstetric fistula in a Ugandan setting. The aim of this study was therefore to identify the risk factors for obstetric fistula from a local context among women in western Uganda.

## Materials and Methods

### *Study setting*

The study was conducted at Kagadi and Kyenjojo general hospitals and Hoima regional referral hospital in western Uganda. The three sites are established sites for fistula outreach treatment. Data was collected from November 2011 to May 2012. This area is a rural, predominantly farming area with poor reproductive health indicators, and have the highest prevalence of fistula (4%) in the country [10]. Maternal health care utilization is low with only 56 per cent of women giving birth assisted by skilled birth attendants [11].

### Study design

A case control study design was used in which 140 cases and 280 controls were included using face-to-face interviews. The interviewers were four research assistants, the first author, who is a gynaecologist trained in fistula surgery and three trained midwives from Mulago and the respective hospitals.

### Study population

Cases were patients confirmed by a doctor to have obstetric fistula irrespective of type and duration. The controls were other women without fistula who had ever given birth and were seeking treatment or attending to patients in the study units. Since these are community units, it was assumed that both cases and controls had a similar environmental exposure and were representative of the population in the area.

### Sample size and sampling

The sample size was calculated *apriori* using OpenEpi, based on the formula described by Kelsey et al [30], with 95% two sided confidence level, 80% power and two controls per case. The exposure factor was the proportion of women delivering with no skilled birth attendant. We assumed that women with fistula were likely to have had no skilled labour monitoring and delivery. We also assumed that the controls were like any other Ugandan women reported in the 2006 Uganda demographic and health survey, where 58 per cent of the women were delivering with no skilled attendance [10]. With the resources at our disposal we aimed to have an effect difference of 14 per cent and hypothetically assumed that the proportion of those with fistula delivering without skilled attendance was 72.4 per cent. From the OpenEpi calculator, our sample size was hence fixed at 140 cases and 280 controls (one case to two controls). Cases were then recruited consecutively and for each case two controls were identified and interviewed.

### Inclusion and exclusion criteria

Women from the study area, who presented for treatment, were screened, confirmed to have obstetric fistula and then enrolled as cases. The cases were confirmed to have obstetric fistula through history and pelvic examination. Controls were women, who had delivered before, with similar or higher parity corresponding to the pregnancy that resulted into fistula in the corresponding case and were frequency age-matched within a range of 5 years. Women with fistula not following labour process or its management like those with carcinoma, trauma, infections and others were excluded. Also women who were not from the study geographical location were excluded as either cases or controls.

### Study variables

The study variables included socio-demographic, physical and obstetric factors highlighted in the literature to predispose women to fistula. The socio-demographic factors were; age at interview, age at marriage, age at first pregnancy, marital status, religion, respondent's education, spouse's education, occupation of spouse, occupation of respondent, and distance to the nearest health facility providing emergency obstetric care including caesarean section. The physical characteristics were height of the respondent and the baby's weight. The obstetric factors were: parity, antenatal care attendance, number of antenatal visits, being accompanied by husband, having a delivery plan, use of herbs in pregnancy and labour, attending antenatal health education classes, and being told the babies presentation. Other obstetric factors included mode of delivery, delivery attendant, and whether there was delay at facility (time spent at the health facility before delivery) or delay in making a decision to seek care, and the duration of labour.

### Data collection

The data were collected using an interviewer-administered questionnaire by the first author assisted by trained research assistants who were midwives. A similar questionnaire was administered to both women who were the cases and those without fistula in the control group. Women who fulfilled the inclusion criteria were interviewed from a quiet and private room identified from outpatient department of the respective hospitals. The interviewers were knowledgeable in the local language and would translate the information and fill the data directly in English. The first author checked that data were filled in before respondents left the study site.

### Data management

All the data were double entered in a computer and cleaned using Epidata version 3.1. Prior to data entry, the Epidata computer screen had been fitted with range and consistency checks. The data were exported to STATA version 12 [31] for further cleaning and then analysed by the first author assisted by the second author.

### Data analysis

All variables were tested for significance at bivariate level using chi-square and the student's t-test for categorical variables and continuous variables respectively. Covariates that were significant at bivariate level with a P-value of less than 0.1 were entered in a multivariate stepwise (backwards and forwards) logistic regression model and the covariates included were tested for interaction and confounding. Odds ratios and 95% confidence interval were computed. The backward likelihood ratio method was used to select the best fitting model. Duration of labour in hours was the most significant variable in the model and was hence taken as the main predictor for obstetric fistula. Interaction terms for duration of labour and other variables were added in the models. We used the log ratio test where the fitness of the model with all the interaction terms included was compared with a fit of the model with none of the interaction terms. During the log ratio tests, the negative two-log likelihood (-2LL) of the full model and the reduced models were compared. Interaction was considered present when the difference between the -2LL were significant at P≤0.5 with a chi square test. Confounding was considered present if the difference between crude and adjusted odds ratios was greater than or equal to ten per cent. Depending on contribution to the goodness of fit of the model, variables left out were brought back into the model. Hosmer and Lemeshow's goodness of fit test was applied to check on quality of the model [32].

### *Ethical considerations*

Respondents were given detailed information about the study: that participation was voluntary, no one would be denied access to services because of refusal to participate in the study, and that information obtained was confidential and would be used only for the purpose of the study. The study received ethical approval from institutional review boards in Uganda and Sweden (requirement for the Makerere University and Karolinska Institutet collaboration). In Uganda, the study received ethical approval from Makerere University, School of Medicine Research and Ethics Committee (#REC REF 2011-104). We also received ethical clearance and approval from the Uganda National Council for Science and Technology (UNCST) registration number HS 1337. We got verbal permission from the respective medical directors/superintendents of Hoima, Kagadi and Kyenjojo hospitals to conduct the study in the respective hospitals. From Sweden, the study protocol was presented and we received approval from the Regional Ethics Committee in Stockholm, (Protocol 2012/2∶4). Informed written consent was obtained from respondents before inclusion in the study. The three participants who were under 18 years assented and also their accompanying parents/guardians gave a written consent. The study conformed to the principles in the Helsinki declaration. All the data were kept confidential and participants were compensated for their time spent during the interviews with 5000 Uganda shillings (USD 2). Those cases not yet operated had their fistulas closed in the week following the interviews by the first author who is trained and skilled in fistula surgery.

## Results

A total of 140 cases and 280 controls fulfilled the inclusion criteria. Of the 280 controls 238 (85%) were patient attendants while 42 (15%) were women seeking care for other gynaecological conditions in the study hospitals. All the women who were approached and fulfilled the inclusion criteria accepted and were interviewed. The respondents were aged 16 to 68 years at the time of the interviews and there were no statistical difference between ages of cases and controls. Of the 140 obstetric fistula cases: 110 (78.6%) had vesicovaginal fistula, five (3.6%) had combined vesicovaginal and rectovaginal fistulas, one (0.7%) had ureterovaginal fistula, 22 (15.7%) had rectovaginal fistula and two (1.4%) had post vesicovaginal fistula repair stress urinary incontinence. A total of 27 (25%) out of 109 women with urogenital fistula had vesicovaginal fistula type 1(VVF I) and ureterovaginal fistula, both of which are considered iatrogenic injuries by the surgeon. The surgeons who had performed the caesarean sections were all general practitioners with a first degree in medicine and surgery. They had also received one year clinical training as interns and were then posted to these hospitals. An obstetrician or a doctor with specialised training performed none of the caesarean sections. There was a high still birth rate among the cases compared with the controls with 100 (71%) of the women with fistula having lost their babies at birth compared to 40 (29%) among the controls (p<0.001). However the risk factors for stillbirth were correlated to the risk for fistula.

### Bivariate results

The details of the socio-demographic characteristics including the P-values, crude Odds ratios and 95% confidence intervals are shown in *table 1*. Being single was significantly associated with developing fistula compared to being married. There was no difference among widowed or divorced compared to married women. Anglicans were less likely to develop fistula compared to women belonging to other religions and so were those respondents who had a higher level of education and/or a spouse with a higher educational level compared to those with no education. Respondents who were income earners were less likely to develop fistula compared to housewives, while being a peasant farmer was more likely to predispose women to getting fistula compared to those with other occupations. Significantly more of the patients with fistula were living far from the nearest comprehensive Emergency Obstetric Care (EmOC) facilities with a median distance of 17.5 km compared to 5 km among the controls. The obstetric and physical factors, which were significantly more likely to predispose women to develop fistula included: Primiparas, use of local herbs in labour, not attending antenatal classes, delivery by caesarean section, women's height of 150 cm or less, delay to decide to seek care, prolonged labour, and delivery of a big baby (3.5 kg or more) as seen in *table 2*. Time spent at the health facility before delivery, delivery attendant, a woman being told the baby's presentation, use of herbs during pregnancy, having a delivery plan, husband accompanying the wife during antenatal care, attending antenatal care services and the number of times a woman attends antenatal care clinic were not associated with fistula in this study.

**Table 1:** Socio-demographic factors of respondents.

Variable	Case n = 140 (%)	Control n = 280 (%)	Crude OR (95% CI)	P-value
**Age at interview**				
10–19	18(12.9)	28(10.0)	1	0.589
20–29	51(36.4)	111(39.6)	0.72(0.36–1.41)	
30–39	49(35.0)	87(31.1)	0.88(0.44–1.74)	
40–49	14(10.0)	40(14.3)	0.54(0.23–1.27)	
50+	08(05.7)	14(05.0)	0.89(0.31–2.54)	
**Age at Marriage**				
<18 years	63(45.6)	127(46.7)	1	0.842
18 years & above	75(54.4)	145(53.3)	1.04(0.69–1.57)	
**Age at first pregnancy**				
<18 years	54(38.6)	118(42.3)	1	0.465
18 years & above	86(61.4)	161(57.7)	1.18(0.77–1.78)	
**Marital status**				
Married	92(65.7)	215(76.8)	1	0.047
Divorced	22(15.7)	33(11.8)	1.56(0.86–2.82)	
Single	21(15.0)	21(7.5)	2.34(1.22–4.48)	
Widowed	05(03.6)	11(3.9)	1.06(0.36–3.14)	
**Religion**				
Anglican	36(25.7)	108(39)	1	0.043
Catholic	63(45.0)	109(39.4)	1.73(1.06–2.83)	
Moslems	08(05.7)	15(5.4)	1.60(0.63–4.08)	
Other	33(23.6)	45(16.3)	2.20(1.22–3.96)	
**Respondent’s education**				
None	32(22.9)	40(14.3)	1	0.001
Primary	87(62.1)	153(54.7)	0.71(0.42–1.21)	
Post primary	21(15.0)	87(31.0)	0.30(0.16–0.59)	
**Spouse's education**				
None	15(10.8)	14(5.0)	1	0.005
Primary	77(55.4)	130(46.4)	0.55(0.25–1.21)	
Post primary	47(33.8)	136(48.6)	0.32(0.15–0.72)	
**Occupation of respondent**				
Housewife	54(38.6)	109(38.9)	1	<0. 100
Peasant farmer	68(48.6)	66(23.6)	2.08(1.30–3.33)	
Income earner	18(12.8)	105(37.5)	0.35(0.19–0.63)	
**Occupation of spouse**				
Peasant farmer	63(45.0)	109(38.9)	1	0.622
Retail business	31(22.1)	66(23.6)	0.81(0.48–1.38)	
Paid employee	21(15.0)	53(18.9)	0.83(0.47–1.47)	
Others	25(17.9)	52(18.6)	0.69(0.38–1.24)	
Median distance to nearest EmOC* unit in Km (Range)	17.5(0.5–130)	5 (0.5–100)	1.03(1.02–1.04)	P<0.001

*EmOC  =  emergence obstetric care.

**Table 2:** Obstetric and physical characteristics of respondents (140 cases, 280 controls).

Variable	Case N (%)	Control N(%)	Crude OR (95% CI)	P-value
**Parity**				
Primipara	46(32.9)	46(16.4)	1	0.001
Para 2–4	47(33.6)	125(44.6)	0.38(0.22–0.64)	
Grand multipara	47(33.6)	109(38.9)	0.43(0.25–0.74)	
**ANC attendance**				
Yes	135(96.4)	275(98.2)	1	0.258
No	05(03.6)	05(01.8)	2.04(0.58–7.16)	
**Number of ANC visits**				
Less than 4	73(54.5)	127(46.4)	1	0.123
4 or more	61(45.5)	147(53.6)	0.72 (0.48–1.09)	
**ANC Husband accompany**				
Yes	60(43.8)	124(45.1)	1	0.803
No	77(56.2)	151(54.9)	1.05(0.70–1.59)	
**Delivery plan**				
Yes	81(58.3)	179(63.9)	1	0.262
No	58(41.7)	101(36.1)	1.27(0.84–1.92)	
**Herbal use in pregnancy**				
Yes	49(35.5)	78(29.0)	1	0.180
No	89(64.5)	191(7.0)	0.74(0.48–1.15)	
**Herbal use in labour**				
Yes	35(25.0)	35(12.5)	1	0.001
No	105(75.0)	244(87.5)	0.43(0.26–0.73)	
**Attend Antenatal classes**				
Yes	106(76.1)	245(87.5)	1	0.003
No	33(23.7)	35(12.5)	2.18(1.29–3.70)	
**Told baby presentation**				
Yes	113(82.5)	243(88.0)	1	0.123
No	24(17.5)	33(12.0)	1.56(0.88–2.77)	
**Mode of delivery**				
Vaginal delivery	71(50.7)	264(94.6)	1	<0.001
Caesarean Section delivery	69(49.3)	15(05.4)	17.10(9.23–31.69)	
**Delivery attendant**				
None	01(00.7)	14(05.0)	1	0.082
Unskilled	20(14.3)	40(14.3)	7(0.86–57.08)	
Skilled	119(85.0)	226(80.7)	7.37(0.96–56.74)	
**Respondent's height (cm)**				
Less or equal 150	46(33.1)	33(11.8)	1	<0.001
More than 150	93(66.9)	247(88.2)	0.27(0.16–0.45)	
**Baby weight**				
Less than 3.5 kg	50(36.2)	163 (58.8)	1	<0.001
3.5 kg and above	88(63.8)	114 (41.2)	1.59(1.29–1.96)	
Time to decide to seek care from onset of labour (hrs); Median (Range)	8(0–72)	4(1–48)	1.06(1.03–1.08)	<0.001
Time at health facility before delivery (hrs); Median (Range)	4(1–72)	4(1–50)	1.09(0.99–1.03)	0.902
**Duration of labour (hrs); Median (Range)**	21(2–96)	12(2–73)	1.06(1.04–1.09)	<0.001

*ANC  =  antenatal care, TBA  =  traditional birth attendant, hrs  =  hours*.

### Multivariate results

All factors, with a p-value ≤0.10 at bivariate analysis, were entered in the model at multivariate analysis. After testing for interaction and controlling for confounding, statistical significant risk factors in the model are presented in *table 3*. The following risk factors were significantly associated with developing a fistula among woman from western Uganda: Caesarean section (adjusted odds ratio [AOR]  = 13.30, 95% CI  = 6.74–26.39), respondent height of 150 cm or less (AOR  = 2.63, 95% CI  = 1.35–5.26), baby weight of 3.5 kg or more (AOR  = 1.52, 95% CI  = 1.15–1.99), prolonged labour (AOR  = 1.06, 95% CI  = 1.04–1.08. Compared to no education, post primary level of education was protective against obstetric fistula (AOR  = 0.31, 95% CI  = 0.13–0.72) and there was no difference between respondents without education and those with primary level education. All other factors, which were significant at the bivariate analysis, were not at multivariate analysis and were therefore excluded from the final adjusted multivariate model.

**Table 3:** Multivariate analysis of risk factors for obstetric fistula.

Variable	Crude OR (95% CI)	Adjusted OR (95% CI)	P-Value
**Duration of labour (hrs)** *	1.06 (1.04–1.09)	1.06 (1.04–1.08)	<0.001
**Baby weight (Kg)** *			
Less than 3.5 Kg	1	1	
More or equal 3.5 Kg	1.59 (1.28–1.95)	1.52 (1.15–1.99	0.003
**Respondent height (cm)** *			
More than 150 cm	1	1	
150 cm or less	3.70 (2.22–6.25)	2.63(1.35–5.26)	0.004
**Mode of Delivery** *			
Vaginal delivery	1	1	
Caesarean Section	17.10(9.23–31.69)	13.30(6.74–26.39)	<0.001
**Respondent's level of education** *			
No education	1	1	
Primary	0.71(0.42–1.21)	0.63(0.31–1.23)	0.176
Post Primary	0.30(0.16–0.59)	0.31(0.13–0.72)	0.007
**Antenatal attendance**			
Yes	1		
No	2.04(0.5–7.16)	-	-
**Distance to the nearest EmOC Obstetric Care unit (Km)**	1.03 (1.02–1.04)	-	-
**Time from on-set of labour to decision to seek care (hrs)**	1.06 (1.03–1.08)	-	-
**Time spent at health facility before delivery (hrs)**	1.09 (0.99–1.03)	-	-

*Significant variables at multivariate analysis, EmOC  =  Emergency obstetric care, hrs  =  hours.

## Discussion

This case control study, which analysed risk factors for obstetric fistula among women in a western Ugandan is the first analytic study conducted in this setting. Previous studies highlighting risk factors for fistula in Uganda were descriptive studies, hence independent association could not be established among the identified factors [27]. In the current study, risk factors for fistula were: delivery by caesarean section, prolonged labour, big baby (weight of 3.5 kg or more), short stature (women with a height of 150 cm or less), and no education or primary education. All other factors that were highlighted in other studies as risk factors for fistula [4], [20], [21], [28], [33]–[36] were not independent risk factors for developing fistula among women in western Uganda after logistic regression analysis.

Age of respondents at interview was similar among cases and the controls because of frequency matching by age. Though age at marriage was similar among the cases and controls, women in this region generally marry at a young age [11]. Almost half of the women were married by 18 years of age, even if the country laws stipulate that marriage should be above 18 years. This low age at marriage is comparable to what has been found in other low income countries like Ethiopia and Ghana where mean age at marriage among women with fistula was 22 and 25 years respectively [35], [37]. However, age at marriage was not significant as a risk factor for fistula at multivariate level in our study.

Marital status was not an independent risk factor for fistula though at bivariate analysis, but more women among controls compared to cases were married. In other countries marital status has been found to be a risk factor but this was not the case in our context [20]. The proportion of fistula patients who were divorced was much lower than in other countries [20], [35], [38], [39]. This could be explained by the culture and religious beliefs in western Uganda whereby society looks at a divorcee as a failure [40]. The alternative explanation could be that the studies elsewhere were descriptive, hence not controlling for confounding and interaction, which was done in this study.

Women's low level of education (none or primary) was a risk factor for fistula, while post primary level of education was protective. A big proportion of both cases and controls generally had none or low-level education (primary). The results agree with what were found in other studies in Africa where no education or low-level education was a significant risk factor for fistula [27], [35], [37]. The fact that there were few educated women with fistula compared to those without has both clinical and policy implications. Clinically women with low-level or no education should be targeted during health education sessions to explain need for delivery under skilled attendance. Policy wise, results from this study show that there is need to target education programs for women to at least post primary level as one of the strategies to control and eliminate obstetric fistula in Uganda.

The risk of developing fistula was not predicted by attending antenatal care or not. Among both cases and controls antenatal attendance was high with more than 96 women in 100 attending at least once. Other studies in Africa have found that failure to attend antenatal care is a risk factor [20], [21], [41]. The finding in our study may be explained by the intense media campaigns in Uganda to end fistula and also prevent maternal morbidity and mortality. A similar trend was observed in a Kenyan study [42]. Despite the fact that more than 96 out of 100 women had attended antenatal care in both cases and controls, the fistula patients took twice as long time to make a decision to seek care compared to the controls. This delay in seeking care may be explained by the fact that despite women attending, the antenatal care messages may not be well packed to emphasize delivery under skilled attendance. This may also be attributed to transport problems, poverty, and home delivery practices [43]. In a study from Entebbe, Uganda, about one in two women who delivered at home with no skilled assistance lacked finances for transport and were poor [43]. This finding is clinically relevant and calls for improvement of quality of information during antenatal care. Policy wise, this calls for government to consider a scheme to fund transport of women in labour and this would prevent women from getting obstetric fistula.

In our study, duration of labour was generally longer among the cases than among controls, with half of the women with fistula having had labour for more than 21 hours (median duration) compared to the controls whose median duration was 12 hours. The results were in agreement with results from a Kenyan study where women who developed a fistula had a median duration of labour of more than 24 hours [42]. This calls for a campaign to intensify labour monitoring using a partograph in Ugandan hospitals and also to encourage women to deliver in hospital. This may be through media campaigns and use of community leaders in identifying and referral of women. Also midwives should receive continuing medical education about the need to monitor the labour process using partograph but even more important what to do when the woman “fall outside” the stipulated progress time.

The baby's weight of 3.5 kg or more was a significant risk factor for fistula. This can be explained by the fact that a big baby is more likely to lead to obstructed labour due to cephalo-pelvic disproportion as has been highlighted in other studies [5], [21], [22], [33]. This calls for vigilant screening for foetal size through well trained/skilled midwives assessing the pelvis and also the use of ultrasound to identify large babies followed by a planned delivery and extra monitoring.

In our study, cases were considerably shorter (height of 150 cm or less) than controls. After controlling for interaction and confounding, women who were 150 cm or less were 2.6 times more likely to develop fistula compared to those whose height was more than 150 cm. The trend can also be explained by the fact that short stature is likely to be associated with high risk of contracted pelvis and cephalo-pelvic disproportion. Similar findings have been found in other studies in Africa [27], [41], [42], [44]. This calls for policies in prevention of obstetric fistula from childhood by addressing nutrition and growth monitoring of the girl child to prevent her from getting stunted. Clinically women of short stature should routinely have pelvic assessment and their labour monitored using a partograph.

In this study, mode of delivery was identified as a risk factor. Women with fistula were more likely to have had delivery by caesarean section compared to controls. Fifty out of 100 women among the cases had had a caesarean section compared to five out of 100 women among the controls. A woman having been delivered by caesarean section was seventeen times more likely to be a case than a control at multivariate analysis. It is known that timely caesarean section by an expert surgeon relieves obstructed labour and hence prevents fistula [5], [12], [14], [33]. However, studies have also demonstrated that fistula actually may be due to caesarean section in women who present late in labour and are operated by physicians without enough surgical experience and with inadequate equipment [35]. The fact that 25 per cent of the women had a fistula whose cause was attributed to injury by surgeon means that caesarean section is not only preventative procedure but can cause obstetric fistula especially the injury of the ureter and the dome of the urinary bladder [35], [45]. It was indeed puzzling to have 25% due to iatrogenic causes with injury to bladder or ureters tied. This however, could be explained by difficult operations or operation in hands of inexperienced surgeon. If these women had not been operated they would most likely have developed fistula but the normal practice would be to have a skilled surgeon whom we never found in these sites. Medical officers with undergraduate training and three months internship training in maternity care run general hospitals in western Uganda. They are posted to these hospitals by ministry of health where they work with no senior surgical supervision. The fact that they encounter women who have delayed at home with an average of 8 hours before making a decision to go to hospital may explain the high prevalence of caesarean related iatrogenic fistulas. The association of fistula to injury by surgeon was also highlighted in a previous study conducted in Uganda [46]. Vesicouterine fistulas are often a result of complications encountered during caesarean section rather than direct consequences of obstructed labour as shown in a study from Ghana where Danso et al [35] found that 16 per cent of the fistulas were due to caesarean section. Another study from Cameroon found similar trends and identified the mode of delivery as a risk factor [47]. However, these patients were found to have supra-trigonal fistula (high vesicovaginal fistula above the point where ureters enter urinary bladder), which tend to be iatrogenic obstetric fistula [21], [33], [47]. This finding has clinical and public health implications whereby there is need to repackage the surgical skills given to new medical officers who do the surgeries in these health centres. This aspect is an important finding from our study, which needs to be addressed. The association of caesarean section and fistula in our study is highly significant, and supported by the types of fistulae found and by other studies. There is therefore need for urgent attention by studying why there is a high prevalence of iatrogenic injuries and appropriate remedial measures put in place. These remedial measures may include retraining of the medical officers, support supervision, timely patient referral and the need for continuous medical education.

### Methodological considerations

Recall bias may distort the associations as cases may have better recalls than the controls. The interviewers were not blinded on who the case or control was but they were trained in order not to influence the answers. Frequency matching means we could not assess the age factor as a risk factor for fistula though other studies have previously highlighted young women to be more likely to develop fistula. The cases of fistula are rather rare and maybe the trend could be different if we were to have large samples. Hospital controls were recruited among women who were sick or attendants to other patients in the hospital and these women might have different exposure histories compared to if we had recruited women from the community, which was not possible due to logistical reasons. The fact that we could not tell which women had cephalo-pelvic disproportion or obstructed labour but no fistula is a limitation. These women should have been excluded since they may be similar to those who developed fistula. Caesarean section for obstructed labour is technically difficult and surgeons require good supervision and training in this procedure. It would have been more meaningful to clarify the nature of these caesarean sections (at what stage of labour were they done/were they elective or emergency). However, we could not get this information and the women were not in position to give reliable information. More clear results from a prospective study would be necessary to solve this limitation. These results are generalizable to western Uganda context based on the fact that we had a reasonable sample size and cases and controls were from the same geographical location. Also the results are generalizable in contexts with similar resource context like western Uganda. However, the findings may not be generalized to other areas with different cultures and settings.

## Conclusions

Caesarean section delivery, prolonged labour, big baby (weight of 3.5 kg or more), respondent height of 150 cm or less (short stature), and low education (no education and primary level) are risk factors for obstetric fistula in western Ugandan. This study demonstrates that in this context there is need to improve on obstetric care and avoid prolonged obstructed labour, improve skills of medical officers and midwives in lower emergency obstetric care units though regular support supervision and in-service training. Since specialists in Obstetrics and gynaecology had operated none of the women, we feel these results may be the basis for retraining of the medical officers (GPs) to gain skills. Antenatal care and proper screening and health education need to be stepped up and be more focused at all levels in the health care system. Women who are short and those whose babies appear big need to be properly assessed and a delivery plan made and implemented in time. The skills of doctors need to be continuously monitored and improved to avoid iatrogenic fistula. Monitoring of labour using a partograph must be revitalized in this setting to avoid prolonged labour. Community interventions geared at making the population aware of the risk factors should be devised.
